# The rules of gene expression in plants: Organ identity and gene body methylation are key factors for regulation of gene expression in *Arabidopsis thaliana*

**DOI:** 10.1186/1471-2164-9-438

**Published:** 2008-09-23

**Authors:** Felipe F Aceituno, Nick Moseyko, Seung Y Rhee, Rodrigo A Gutiérrez

**Affiliations:** 1 Departamento de Genética Molecular y Microbiología, Pontificia Universidad Católica de Chile, Avenue Libertador Bernardo O'Higgins 340, Santiago, Chile; 2 Department of Plant Biology, Carnegie Institution of Washington, 260 Panama St, Stanford, CA, 94305, USA; 3 Department of Biology, New York University, 100 Washington Square East, 1009 Main Building, New York, NY 10003, USA

## Abstract

**Background:**

Microarray technology is a widely used approach for monitoring genome-wide gene expression. For Arabidopsis, there are over 1,800 microarray hybridizations representing many different experimental conditions on Affymetrix™ ATH1 gene chips alone. This huge amount of data offers a unique opportunity to infer the principles that govern the regulation of gene expression in plants.

**Results:**

We used bioinformatics methods to analyze publicly available data obtained using the ATH1 chip from Affymetrix. A total of 1887 ATH1 hybridizations were normalized and filtered to eliminate low-quality hybridizations. We classified and compared control and treatment hybridizations and determined differential gene expression. The largest differences in gene expression were observed when comparing samples obtained from different organs. On average, ten-fold more genes were differentially expressed between organs as compared to any other experimental variable. We defined "gene responsiveness" as the number of comparisons in which a gene changed its expression significantly. We defined genes with the highest and lowest responsiveness levels as hypervariable and housekeeping genes, respectively. Remarkably, housekeeping genes were best distinguished from hypervariable genes by differences in methylation status in their transcribed regions. Moreover, methylation in the transcribed region was inversely correlated (R^2 ^= 0.8) with gene responsiveness on a genome-wide scale. We provide an example of this negative relationship using genes encoding TCA cycle enzymes, by contrasting their regulatory responsiveness to nitrate and methylation status in their transcribed regions.

**Conclusion:**

Our results indicate that the Arabidopsis transcriptome is largely established during development and is comparatively stable when faced with external perturbations. We suggest a novel functional role for DNA methylation in the transcribed region as a key determinant capable of restraining the capacity of a gene to respond to internal/external cues. Our findings suggest a prominent role for epigenetic mechanisms in the regulation of gene expression in plants.

## Background

Understanding the regulation of gene expression is essential to understand the form and function of living systems. Microarray technology has been widely used in many organisms to understand genome-wide changes in gene expression in response to treatments [1], in different organs [2], cell-types [3] and along developmental time series [4]. Therefore, a large amount of microarray data representing many different biological conditions has accumulated over recent years. This data has been used successfully to hypothesize on gene function on a global scale in different organisms, such as yeast and *C. elegans *[5-7], and to suggest shared regulatory mechanisms. Promoters of genes with strongly correlated expression patterns in multiple experiments are likely to be bound by a common transcription factor [8], and conserved regulatory motifs have been identified based solely on expression data [9]. From a systems view, however, we believe that this data has been underutilized as a resource to understand the basic rules of gene expression.

To learn the general rules that govern gene expression in plants, we took advantage of a large microarray database available for Arabidopsis in the NASCarrays database [10]. Using this data, we defined the internal and external cues that regulate the expression of all of the Arabidopsis genes that are represented in the Affymetrix ATH1 gene chips. We quantified the effect of the different experimental conditions on gene expression, which revealed tissue type to be the most influential variable. We also analyzed different structural features and correlated it with the capacity of the genes to respond to the different stimuli. We found evidence for a mechanistic relationship between DNA methylation in the body of the gene (i.e., the transcript region) and the regulation of gene expression, thus assigning a novel and important role for the methylation of the body of the gene in eukaryotic genomes.

## Results and discussion

### The Arabidopsis transcriptome is robust to most perturbations but strongly influenced by organ type

**Figure 1:**
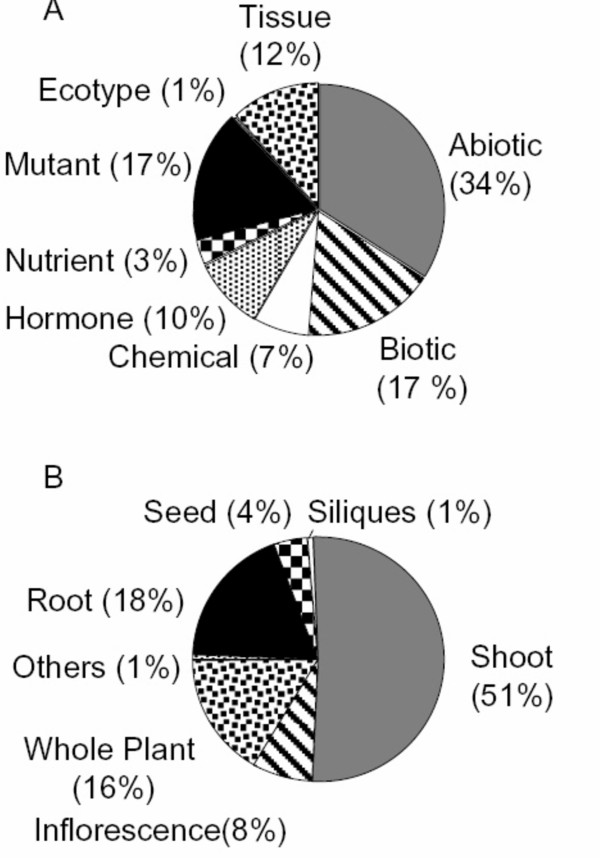
**Classification of experiments from the NASCarrays database**. Pie charts with the classification of microarray experiments according to the experimental factor categories defined by TAIR (A) or the organ used to extract RNA to perform the microarray experiments (B).

In an effort to discover new principles that govern gene expression in *Arabidopsis thaliana*, we integrated and analyzed publicly available whole-genome microarray data for this model plant. From this data, we defined 474 biologically relevant comparisons (i.e. control vs. treatment) as described in Materials and Methods (Additional File 1). These comparisons spanned a wide variety of experimental conditions and plant organs (Figure 1). We wished to evaluate the effect of the different experimental factors that defined each comparison on genome-wide gene expression patterns. To do so, we defined differential gene expression using the RankProducts method [11]. This method outperformed other methods to determine regulation of gene expression in previous studies [11,12] and in our own evaluation (see Materials and Methods), particularly in datasets with a small number of replicates.

**Figure 2:**
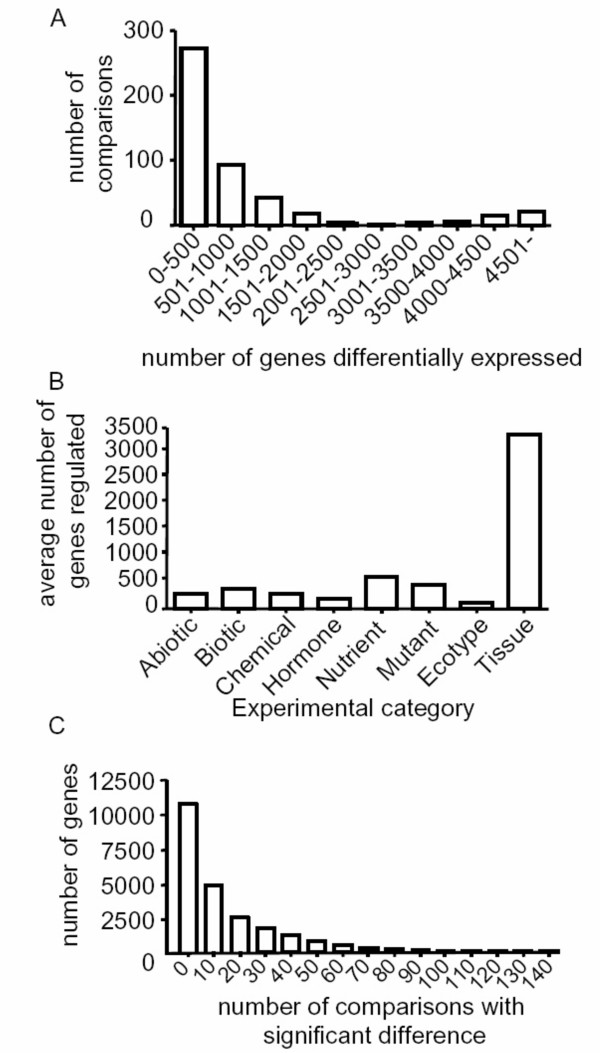
**Global characteristics of the Arabidopsis transcriptome**. A) Histogram of the number of genes (X-axis) regulated in a given number of comparisons (Y-axis). B) Average number of genes regulated by each experimental category as defined in Figure 1A. C) Histogram of the number of comparisons (X-axis) for which the specified number of genes (Y-axis) show significant regulation.

We first examined the number of differentially regulated genes per comparison. We found their distribution to be far from normal. As shown in Figure 2A, some comparisons exhibit more than 4,000 differentially expressed genes. These outliers were exclusively comparisons between different organs. In fact, organ type was the strongest experimental factor contributing to the number of differentially expressed genes. Other experimental factors, regardless of their nature, showed an approximately 10-fold smaller impact on gene expression with an average of 337 genes regulated per comparison (Figure 2B). Moreover, approximately 10% of the Arabidopsis genes did not respond to any of the stimuli in the dataset and were only differentially expressed between organ samples. Thus, organ is by far the most important factor in determining genome-wide expression levels. Furthermore, the upper 5^th ^percentile (ordered by the number of genes regulated) of the 77 mutant vs wt comparisons involved only genes whose mutations have well documented developmental phenotypes. These genes were AP2-6[13], ARR21[14], GLABROUS1[15] and LFY-12 mutations [16]. They regulated 1475, 1420, 1379 and 1362 genes, respectively – a much more than the category average (471 genes). These results indicate that global gene expression patterns are established during plant development. The results also suggest that the Arabidopsis transcriptome is robust to most perturbations, with only an estimated 1.5% of the genome on average responding in a single experiment to experimental factors such as chemical or hormone treatments, pathogen challenges or environmental stress. A detail of the categories in which each of the Arabidopsis genes responds is presented in Additional File 2. Additional Files 3 to 10 contain the genes that respond in exclusively one category, including organ type.

Given its impact on global gene expression levels, we next wished to evaluate the importance of organ type in the context of typical experimental factors that are tested in the laboratory. We compared the number of genes responding in shoots or roots for each of the nine treatments in the AtGenExpress abiotic stress series. On average, only 13% of the total genes that responded to a treatment responded in both organs. By contrast, a much higher proportion of genes (88%) were regulated by the treatment in an organ-specific manner (Additional File 11). This data indicate that plant responses to external stimuli are strongly organ-dependent and underscore the need for a more thorough survey of organ-specific and, by extension, cell-specific responses in Arabidopsis and other plants [3].

### Housekeeping and hypervariable genes possess marked structural differences

To identify properties that explain the capacity of a gene to respond to stimuli, we ranked genes based on the number of comparisons in which they are differentially expressed. As shown in Figure 2C, the Arabidopsis genome contains genes that are regulated in a wide range of comparisons, with an average of 14 comparisons, or 3% of the total comparisons in our dataset. The underlying data is provided in Additional File 12. We expect structural differences to be maximized at the extremes of this distribution. We defined housekeeping genes based on three criteria: (1) genes that were not differentially expressed in any of the 474 comparisons, (2) genes with signal intensities higher than the median intensity across the entire dataset and (3) genes with the lowest signal variability (measured with the interquartile range, see Materials and Methods) across the entire dataset. In contrast, we defined hypervariable genes based on the following three criteria: (1) genes that were within the top 1% of the gene responsiveness distribution, (2) genes with the largest signal variability, and (3) genes that show differential expression by stimuli from six of the eight categories described in Figure 1A. These criteria defined 384 housekeeping genes and 123 hypervariable genes (Additional files 13 and 14).

A previous study positively correlated expression levels with gene size in plants [17]. To understand how gene responses to stimuli relate to gene size and other structural features, we analyzed the structure of housekeeping and hypervariable genes. Housekeeping genes were significantly larger and had more introns than do hypervariable genes and were above genome averages for both criteria (Table 1). By contrast, hypervariable genes were significantly shorter and contained fewer introns than average (Table 1). Interestingly, a functional annotation of the hypervariable gene set indicates that it is enriched for genes involved in responses to internal and external stimuli (Additional File 15). Most hypervariable genes were plant specific as defined in a previous study [18], and the set was enriched for genes that code for unstable transcripts [19] (Table 1). These results suggest that plants favored the evolution of small, hypervariable genes to respond quickly and economically to multiple environmental signals.

**Table 1:** Contrasting features of housekeeping and hypervariable genes.

**Gene feature**	**Housekeeping**	**Hypervariable**	**Genome**
**CDS length (bp)**^*a*^	2624 (s.e. = 89)	1178 (s.e. = 73)	1931 (s.e. = 8)
**Gene length (bp)**^*a*^	3117 (s.e. = 87)	1493 (s.e. = 78)	2229 (s.e. = 8)
**Total exon length (bp)**^*a*^	1941 (s.e. = 52)	1169 (s.e. = 50)	1568 (s.e. 6)
**Total intron length (bp)**^*a*^	1173 (s.e. = 52)	323 (s.e. = 44)	660 (s.e. = 4)
**Number of exons (pb)**^*a*^	8 (s.e. = 0.31)	3 (s.e. = 0.24)	5 (s.e = 0.03)
**Genes without introns**	6% (p = 5E-16)	33% (p = 0.0007)	28%

**Average number of transcription factor binding sites**^*b*^	27 ± 1.2 (p < 0.01)	46 ± 1.8 (p < 0.0001)	30 ± 0.1

**TATA-containing genes**^*c*^	5% (p = 1.3E-6)	45% (p = 6.1E-15)	15%

**Genes coding for unstable transcripts**^*d*^	0% (n.a.)	8% (p = 9E-11)	1%

**Shared among eukaryotes**^*e*^	18% (p = 0.002)	7%	14%
**Plant-specific**^*e*^	11%	34% (p = 2E-10)	14%

**Body methylation**^*f*^	63% (p = 1.5E-35)	8% (p = 2E-10)	34%
**Promoter methylation**^*f*^	3%	3%	5%
**Body methylation**^*g*^	36% (p = 9.1E-21)	2% (p = 3.8E-8)	20%

The first column lists various features analyzed for housekeeping genes (second column), hypervariable genes (third column) and the whole genome (fourth column). Rows report average and standard error or percentage values. P values for significant (p < 0.01) enrichment or depletion as compared to the genome occurrence are shown in parenthesis. *a*, differences between all groups are significant (p < 0.01) as determined by ANOVA.*b*, average number of cis-acting regulatory elements as defined in the AGRIS database [47]. p-value was determined by a t-test. *C*, presence of TATA-box as determined by the MotifSearch algorithm [26]. Similar results were obtained with an alternative TATA-box definition [27]. *d*, unstable transcripts as defined in [19]. *e*, phylogenetic profiles as defined previously [18]. Only significantly enriched profiles are listed. *f*, methylation patterns as determined in [24]. *g*, methylation patterns as determined in [25]. n.a., not applicable.

Eukaryotic genes are transcriptionally regulated by the coordinated interaction of multiple protein factors that interact with discrete binding sites and with each other [20]. These binding sites are usually located upstream of the transcribed region they regulate [20]. The promoters of hypervariable genes often have a TATA-box sequence and contain a larger number of predicted transcription factor binding sites as compared to the housekeeping genes or the genome average (Table 1 and Additional File 16). These data suggest that the presence of a TATA box and the number of transcription factor binding sites in the promoter region of some of the most responsive genes in Arabidopsis may explain their capacity to respond to stimuli, as was previously found in an analysis of a smaller expression dataset [21]. However, it is clear that this simple rule does not always apply and that other factors are necessary to explain gene expression responses.

In addition to gene structure, epigenetic mechanisms such as DNA methylation are known to have an impact on gene expression in eukaryotes, particularly in heterochromatic regions [22,23]. To evaluate the potential role of DNA methylation in the gene expression responses observed for housekeeping and hypervariable genes, we analyzed the methylation patterns of these two groups of genes. We used two recently published genome-wide methylation data sets [24,25] to analyze methylation in the promoter and transcribed regions of each gene. Using the methylome data produced by Zhang et al. [24], we found that a large proportion of housekeeping genes were methylated in their transcribed regions (a significant enrichment compared to the expected genome frequency; p = 1.5E-35, Table 1). By contrast, only 8% of the hypervariable genes were methylated in their transcribed regions (a significant depletion; p = 2E-10, Table 1). Similar results were obtained with an independently generated methylome data set [25]. These results suggest that the capacity of Arabidopsis housekeeping and hypervariable genes to respond to stimuli not only depends on structural features in their promoter or transcribed regions, such as transcription factor binding sites, but may also have an important epigenetic component.

### Transcript region methylation is the most important factor to explain genome-wide responses to internal/external stimuli

To evaluate the importance of these features for gene expression responses on a genomic scale, we performed a regression analysis of the gene responsiveness for all Arabidopsis genes as a function of each of the structural features described above. We used a linear model of the form: Y ~ αX + β, where Y was the observed gene responsiveness of all genes and X was the structural feature under evaluation (e.g. presence of TATA-box, cis-acting binding sites in the promoter or gene body methylation). Thus, the effects detected were free from any bias arising from gene selection, as could be the case when analyzing this relatively small group of housekeeping and hypervariable genes.

**Figure 3:**
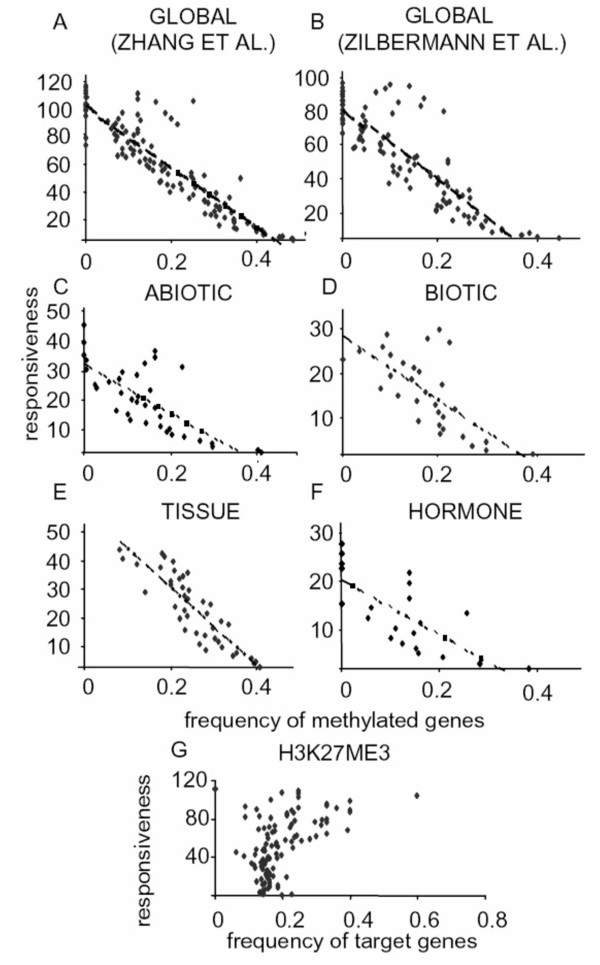
**Correlation between methylation and gene responsiveness**. (A) Plot of the frequency of methylated genes (according to Zhang et al. [24]; X-axis) within a group of genes against the number of comparisons in which that group of genes is regulated (Y-axis). The dotted line represents the regression line. B) Same as (A) except using data from Zilberman et al [25]. C) to E). Same as (A) except with the different experimental categories defined in Figure 1A, using methylome data from Zhang et al [24]. G) Same as (A) except the X-axis represents the frequency of genes that are the target of trimethylation on H3K27 [30].

Notably, using the two independently generated methylome datasets [24,25], gene responsiveness showed a remarkably high negative correlation with the presence of methylation in the transcribed region of the gene. Both datasets generated models with a coefficient of determination (R^2^) of 0.8 (share of explained variability, Figure 3A–B). A similar result was obtained using average fold-change ≥ |2| (treatment versus control) as a criterion to determine gene responsiveness (Additional Files 17 and 18). This correlation was independent of the type of experimental factor, as similar trends were observed when analyzing each experimental category individually for both methylome datasets (Figure 3C–F and Additional File 19). Next, to transcript region methylation, the presence of a TATA-box was the second best factor to explain gene responsiveness, and it had a positive effect. R^2 ^for two definitions of TATA-box [26,27] were 0.49 or 0.68. Two factor models that included transcript region methylation and the presence of a TATA-box slightly improved the R^2 ^over those obtained with methylation alone (Table 2). Two factor ANOVA models (Additional File 20) confirmed the stronger effect of gene body methylation on responsiveness, as determined by the Tukey comparison procedure [28]. However, goodness of fit estimation by the Bayesian Information Criteria [29] suggests that additive models, including TATA-box and methylation, are better than one-factor ANOVA models. (Additional File 20). Interestingly, this also suggests that the effect of TATA-box and methylation are independent, as interaction terms are not significant in these models (not shown). None of the other structural features (gene size, presence of introns, number of binding sites, etc) yielded models with such high R^2 ^on a genomic scale. Thus, gene body methylation and, to a lesser extent, TATA-box presence explained gene responsiveness on a global scale. It is not possible, however, to infer from this data the mechanistic relationships between TATA-related factors, gene body methylation status and regulation of gene expression.

**Table 2:** Results of the simple and multiple linear regression analyses

**Explanatory variable(s)**	**Data Source**	**r**^2^	**p**	**Coefficient**
**Methylation frequency**	[24,25]	0.8	<2E-16	n.r.
		0.8	<2E-16	n.r.

**Frequency of genes target of H3k27me3**	[30]	0.12	0.000207	n.r.

**Gene size**	TAIR Genome v6.0	0.02	>0.01	n.r.

***Cis*-acting elements**	[48]	0.05	>0.01	n.r.

**TATA-box frequency**	(MotifSearch, [26]) (PlantProm, [26])	0.49 0.68	<2E-16 <2E-16	n.r. n.r.

**Methylation + TATA-box**	[24]+ (MotifSearch, [26])	0.84	<2E-16^*a*^ 0.0002^*b*^	-201.5^*a*^ 35^*b*^
	
	[24] + (PlantProm, [26])	0.86	<2E-16^*a*^ 1.00E-09^*b*^	-168^*a*^ 50.5^*b*^
	
	[25] + (MotifSearch, [26])	0.87	2.00E-16^*a*^ 5.00E-09^*b*^	-158.6^*a*^ 54.8^*b*^
	
	[25] + (PlantProm, [26])	0.84	<2E-16^*a*^ 0.0006^*b*^	-194.3^*a*^ 39^*b*^

Column 1 reports the explanatory variables used to model gene responsiveness. Column 2 indicates the source of the data (reference). Columns 3 and 4 report the different statistics obtained with the linear regression. n.r., not reported; n.d., not determined. *a*, statistics for methylation variable. *b*, statistics for TATA-box variable. Column 5 shows the coefficients from the linear regression analysis.

**Figure 4:**
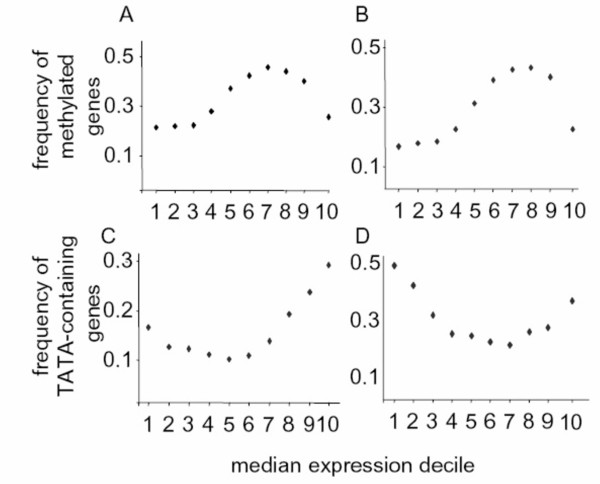
**Lack of linear correlation between expression levels and gene body methylation or TATA-box presence**. (A) Plot of the median expression level across the whole NASC arrays dataset in 10% bins (X-axis) versus the frequency of methylated genes in the bin (Y-axis), as determined by Zhang et al. [24]. (B) Same as (A), except using data from Zilberman et al. [25]. C) Same as (A), except the Y-axis represents the frequency of TATA-containing genes according to the MotifSearch definition [26]. D) Same as (C), but using the PlantProm definition [27].

The effect of DNA methylation on gene responsiveness could be explained by a simple transcriptional gene silencing effect [22,23]. Silencing a gene would render it unable to be regulated. If so, transcript region methylation should correlate with expression levels. Comparing the frequency of methylation to the median expression level of the whole dataset revealed no such trend (Figure 4). The most and the least highly expressed genes are likely to lack methylation within their body, as previously reported [25]. Similarly, no correlation was found between the presence of a TATA-box and gene expression levels. (Figure 4). Moreover, no relationship was evident between expression level and gene responsiveness in our data set (Additional File 21).

We also evaluated the relationship between the presence of modified histones and gene responsiveness. We used a recently published genomic survey of trimethylation in lysine 27 of histone H3 (H3K27me3) f[30]. We found a weak correlation between the frequency of H3K27me3 gene targets and gene responsiveness, with an R^2 ^of 0.12 (Figure 3F and Additional File 19). This finding is consistent with the hypothesis that H3K27me3 mostly acts in a DNA methylation-independent manner, as previously suggested [30]. Other histone modifications, such as H3K4 or H3K9 methylation [31] or combinations thereof [32], may be related to gene body methylation in Arabidopsis, thus "marking" the corresponding chromatin region for or against the regulation of gene expression [33].

### Gene body methylation and regulation of expression by nitrate in TCA cycle genes

As a case-study and to provide a concrete example of the influence of methylation patterns on the regulation of gene expression, we focused on a discrete biological process and experimental factor: nitrate. Nitrate has been shown to be a signal to regulate gene expression in plants [34]. We chose four microarray experiments in which wild-type seedlings were treated with different nitrate concentrations. These nitrate experiments were not included in the microarray database used in the previous sections. We found that nitrate regulates many genes in central metabolic pathways such as the TCA cycle [34-37]. We analyzed responsiveness and nitrate regulation for all genes coding for TCA cycle enzymes. Most of the genes (29 out of 36, data not shown) did not respond to the nitrate treatments, as expected due to the robustness of expression patterns in Arabidopsis (see Figure 2B). Among the genes regulated by nitrate, we found a malate dehydrogenase gene (MDH, At3g47520), two genes coding for NAD^+ ^dependent isocitrate dehydrogenases (At5g03290 and At4g35260) and a putative NADP^+ ^dependent isocitrate dehydrogenase (At1g65930) (Table 3). Remarkably, these four genes were classified as unmethylated in studies by both Zhang et al. [24] and Zilberman et al. [25]. Moreover, body methylated genes were enriched among the analyzed genes that were not regulated by nitrate (Table 3). For instance, among eight genes coding for malate dehydrogenase that are not regulated by nitrate, five are methylated according to the two methylome datasets. This is a much higher frequency than is expected by chance (p < 0.05), as only 20–34% of the genes were methylated according to the two methylome datasets. The same was true for the isocitrate dehydrogenases, with enrichment of methylated genes for those that did not respond to the nitrate treatment (p < 0.05). These results agree with the proposed relationship between gene body methylation and the regulation of gene expression in response to regulatory signals (in this case, nitrate). Moreover, it suggests gene body methylation plays a role in the regulation of gene expression in physiological processes such as the reprogramming of carbon metabolism in response to nitrogen nutrient availability [38].

**Table 3:** Relationship between the methylation status and nitrate regulation of TCA cycle genes.

AGI number	Gene Annotation	Responsiveness to nitrate	Methylation status^a^
At3g47520	MDH (malate dehydrogenase); malate dehydrogenase	3	U
At1g04410	malate dehydrogenase, cytosolic, putative	0	A
At1g53240	malate dehydrogenase (NAD), mitochondrial	0	M
At2g22780	PMDH1 (PEROXISOMAL NAD-MALATE DEHYDROGENASE 1); malate dehydrogenase	0	M
At3g15020	malate dehydrogenase (NAD), mitochondrial, putative	0	U
At5g09660	PMDH2 (PEROXISOMAL NAD-MALATE DEHYDROGENASE 2), PMDH2 (PEROXISOMAL NAD-MALATE DEHYDROGENASE 2); malate dehydrogenase	0	M
At5g56720	malate dehydrogenase, cytosolic, putative	0	M
At5g58330	malate dehydrogenase (NADP), chloroplast, putative	0	M
At5g43330	malate dehydrogenase, cytosolic, putative	0	U
At5g03290	isocitrate dehydrogenase, putative/NAD+ isocitrate dehydrogenase, putative	2	U
At4g35260	IDH1 (ISOCITRATE DEHYDROGENASE 1); isocitrate dehydrogenase (NAD+)	2	U
At1g65930	isocitrate dehydrogenase, putative/NADP+ isocitrate dehydrogenase, putative	1	U
At3g09810	isocitrate dehydrogenase, putative/NAD+ isocitrate dehydrogenase, putative	0	M
At4g35650	isocitrate dehydrogenase, putative/NAD+ isocitrate dehydrogenase, putative	0	U
At5g14590	isocitrate dehydrogenase, putative/NADP+ isocitrate dehydrogenase, putative	0	M
At1g54340	ICDH (ICDH); isocitrate dehydrogenase (NADP+)	0	M

This table provides the AGI number, the gene annotation, regulation by nitrate as determined from four independent experiments (see main text) and the methylation status according to the two methylome datasets used in this work. This table includes all the different malate dehydrogenase and isocitrate dehydrogenase isozyme-coding genes present in the Arabidopsis genome, according to VirtualPlant . ^a^Methylation code: U, unmethylated in both datasets; M, methylated in both datasets; A, ambiguous according to Zilberman et al. [25] but unmethylated according to Zhang et al. [24].

## Conclusion

The analysis of the large and heterogeneous whole-genome microarray dataset available in the public domain proved useful to evaluate principles that govern regulation of gene expression in plants. Our global and systematic analysis of the quantitative effect of different experimental factors (e.g., mutations, stress and organ identity) on the plant transcriptome revealed the key role of developmental processes for establishing mRNA levels throughout the plant. This process in turn determines how cells, organs and tissues respond to exogenous cues. Our data indicate that plant responses to external stimuli are strongly organ-dependent and underscore the need for a more thorough survey of organ-specific and, by extension, cell-specific responses in Arabidopsis and other plants [3].

The second part of our analysis provided a weighted insight into the role of different molecular mechanisms in the global regulation of gene expression in Arabidopsis. The data indicate that DNA methylation within the body of Arabidopsis genes is a key factor that may determine or negatively influence the capacity of genes to respond to internal or external cues. The presence of a TATA-box may favor gene responsiveness but to a lesser extent than the negative effect of DNA methylation. Surprisingly, our data indicate that other gene structural features (e.g., number of cis-acting elements, gene size, presence and number of introns) are less important than DNA methylation and the presence of a TATA-box. These results highlight the importance of epigenetic mechanisms for the global control of gene expression. As a concrete example, we found consistency between regulation by an external stimulus (nitrate) and gene body methylation for a discrete biological process, the TCA cycle, beyond what would be expected by chance. The results presented here suggest a model whereby gene body DNA methylation restrains the ability of a gene to be regulated, regardless of regulatory signals (e.g., binding sites for specific transcription factors in the promoter region). This effect would not be directly dependent on basal gene expression levels. Moreover, our results provide a plausible functional role for the DNA methylation that is found in the body of a large number of Arabidopsis genes. This new role differs from the proposed role for DNA methylation in suppressing spurious transcriptional initiation [25,39] and reinforces the link between the regulation of gene expression and DNA methylation in eukaryotes.

## Methods

### Data processing

The CEL data files comprising all ATH1 Affymetrix hybridizations through the end of 2005 were obtained from NASCArrays through the AffyWatch Subscription Service. This data comprised 1887 hybridizations corresponding to 108 different experiments. The entire hybridization set was normalized using the Robust Multiarray Analysis method [40] available from Bioconductor . Once normalized, the hybridizations were quality-controlled using the method devised by Persson et al [41]. Briefly, this method uses a Kolmogorov-Smirnov goodness-of-fit test to evaluate whether the distribution of deleted residuals for an individual hybridization deviates from a "t" distribution. According to Persson et al [41], this occurs when the value of the *D *statistic from the goodness-of-fit test is more than 0.15. The CEL files with a *D *statistic over this cut-off value were excluded from the analysis. This step resulted in the exclusion of 186 CEL files.

For the analysis of differential expression, the remaining 1701 hybridizations were mapped to their corresponding experiments. Controls and biologically meaningful tests were identified and grouped with their replicates. Comparisons in which the control or treatment hybridizations had less than 2 replicates were discarded. This process resulted in a list of 474 biologically meaningful comparisons (control versus test), including 1295 hybridizations. In the case of tissue comparisons, we used rosette leaves as a control, and all other tissues were considered tests. Rosette leaves were chosen as the reference because they are the prototypical organ system [2]. We classified the comparisons according the experimental variable involved using the criteria defined by TAIR [42], and according to the RNA source organ (Figure 1)

### Differential expression analysis

The comparisons were analyzed for differential gene expression using the RankProducts method [11], implemented as a Bioconductor package [43]. This method outperformed other methods to define differential expression in a study comparing ten different methods [12], particularly in high-noise, low-replicate datasets. Our comparisons have a low number of replicates (average = 2.7) and a high variability (pooled variance of the whole dataset = 4.04). We also evaluated the performance of RankProducts as compared to other popular alternative methods based on biological criteria. We defined regulation using RankProducts, average fold change and t-test with different FDR corrections for multiple testing [44,45]. To evaluate the methods, we randomly chose five test comparisons from different experimental categories (e.g. biotic, abiotic, tissue).

We evaluated the functional coherence of the differentially expressed genes by the different methods by evaluating enriched gene ontology (GO) terms in the resulting lists. For most of the comparisons tested, visual inspection revealed enriched GO terms that were obviously related to the experimental factor. This was not the case for the other methods. As an example, 245 genes were found to be differentially expressed in the comparison DO.1.1 (Additional File 1). Out of these 245 genes, 217 were previously identified as regulated in these experiments using a different method in a prior study [46]. In addition, the 140 down-regulated genes determined by RankProducts showed an overrepresentation of "transport" and other functional terms previously known to be related to the experimental factor [46]. Similarly, the abscisic acid response evaluated in comparison AQ.4.4 (Additional File 1) identified 241 differentially expressed genes. Among the up-regulated genes, we found that the 'abscisic acid response' functional term was overrepresented.

With the results of the differential expression analysis, a "regulation matrix" was created. This matrix contained the p-value for the down- and up-regulation of all of the ATH1 Affymetrix chip probes across the 474 comparisons. The cut-off for defining a probe as differentially expressed was 0.05. The complete data file with ratios is available from . Additional data files are available upon request.

### Housekeeping and hypervariable gene definition

The least responsive genes (housekeeping genes) were defined as follows: first, we selected genes which did not show differential expression in any comparison (5652 genes). Second, these genes were filtered for expression above the median of the entire NASC dataset (1758 genes). Third, we choose only those having a signal difference between the 1^st ^and 3^rd ^quartile (interquartile range) that was in the bottom 5 percentile of the signal interquartile ranges from the whole dataset. This ensured the selection of 384 expressed Arabidopsis genes that exhibit the lowest expression variability.

For the most responsive genes (hypervariable genes), we first choose genes that were regulated in 86 or more comparisons, corresponding to the top 1% most responsive genes from Figure 2C. Second, we selected genes that were regulated in at least six out of the eight categories defined in Figure 1A to avoid any bias due to large categories (e.g., abiotic stress experiments). We did not use an expression cutoff, since as expected hypervariable genes were sufficiently expressed, with a median signal of 8.4 across the NASC dataset (the global median is 7.4). From the 185 genes selected by these criteria, we choose those with a signal interquartile range in the upper 5% of the entire dataset. Thus, we defined a group of 123 "hypervariable genes".

### Structural and phylogenetic analyses and correlation with gene responsiveness

Gene structural features (gene, CDS, exon, intron lengths and numbers) – were obtained from the TAIR 6.0 Arabidopsis genome [42]. Phylogenetic classifications of the genes were obtained from the Plant-Specific Database [18]. Methylation status of the different genes (body methylated, body unmethylated and promoter methylated) was obtained from Zhang et al. [24] or Zilberman et al. [25]. TATA-box presence or absence in the promoter region of Arabidopsis genes was obtained from Molina and Grotewold[26]. The number of transcription factor binding sites in gene promoters was calculated from the data in the AtCis Database from AGRIS [47]. Unstable transcripts were extracted from the data generated by Gutierrez et al. [19]. All data were processed using custom-made scripts in R  and Perl languages. Statistical analyses and graphs were done in R, GraphPad Prisma 4.0 software or Microsoft Excel.

### Statistical and regression analysis

Calculation of significant enrichment or depletion was done in R using the hypergeometric distribution. t-tests were carried out with the GraphPad Prisma 4.0 software. Simple and multiple linear regression models used to predict gene responsiveness as a function of various structural parameters were done in R. We used simple models of the form: *Y *~ α*X *+ β, where *Y*, the response variable, is the gene responsiveness and *X *is the value of the structural feature under evaluation. In the case of categorical features, such as methylation or the presence of TATA-box, *X *represented the frequency of the feature in a group of genes sharing the same responsiveness. For multiple linear regressions, we used models of the form: *Y *~ α*X *+ β*Z *+ γ*W*... where *Y *was the gene responsiveness and *X*, *Z*, *W*, etc. corresponded to different features to evaluate. Models were fitted using the lm function from the R statistical software. We used the R^2 ^parameter to evaluate the quality of the model, since R^2 ^represents the extent of data variability explained by the model. As a complementary approach for categorical features, we used one factor ANOVA models. They have the form *Y *~ α*X *+ β, where *X *was a factor encoding the presence or absence of those features at two different levels. We used the 'aov' function in R to fit the model. We used the F statistic to estimate the significance of the contribution of the factors to the response. To estimate the differences between the levels of the factors, we followed the Tukey procedure, using the 'glht' function from the 'multcomp' package in R. The Bayesian Information Criteria was calculated in R using the 'BIC' function in the package 'nlme'. Graphs were done in R, GraphPad Prisma 4.0 software or Microsoft Excel.

### Gene body methylation and regulation by nitrate for TCA cycle genes

We retrieved the genes corresponding to the TCA cycle from AraCyc [48]. We then determined the gene responsiveness of these genes in four previously published microarray data sets [34-37] that were not included in the NASCarrays database and were therefore not used to derive our genome-wide conclusions. We intersected the methylation status [24,25] and regulation by nitrate of the genes encoding malate dehydogenases and isocitrate dehydrogenases using the VirtualPlant software platform . Statistical analysis of enrichment was performed as described above.

## Authors' contributions

FFA carried out the bioinformatics and statistical analyses and wrote the manuscript. NM and SYR revised the manuscript critically for important intellectual content. RAG carried out some of the bioinformatics analyses, wrote the manuscript and was responsible for the conception of the study, the design of the data analysis and the interpretation of the results. All authors read and approved the final manuscript.

## Supplementary Material

Additional File 1 **Control vs. tests comparisons**. List of the analyzed 474 comparisons in the NASCarrays database, annotated according to the experimental factor and plant structure categories. NASC experiment numbers are provided. Click here for file

Additional File 2 **Gene responsiveness by categories**. Table detailing the number of experiments, within the eight experimental categories, in which each Arabidopsis gene is regulated. The number in parenthesis in the header of the Table indicates the total number of experiments in each category. Click here for file

Additional file 3 **Genes regulated specifically in one experimental category**. Each file provides the individual genes responding exclusively in abiotic, biotic, ecotype, chemical, hormone, mutant, nutrient or organ comparisons, respectively. Click here for file

Additional file 4 **Genes regulated specifically in one experimental category**. Each file provides the individual genes responding exclusively in abiotic, biotic, ecotype, chemical, hormone, mutant, nutrient or organ comparisons, respectively. Click here for file

Additional file 5 **Genes regulated specifically in one experimental category**. Each file provides the individual genes responding exclusively in abiotic, biotic, ecotype, chemical, hormone, mutant, nutrient or organ comparisons, respectively. Click here for file

Additional file 6 **Genes regulated specifically in one experimental category**. Each file provides the individual genes responding exclusively in abiotic, biotic, ecotype, chemical, hormone, mutant, nutrient or organ comparisons, respectively. Click here for file

Additional file 7 **Genes regulated specifically in one experimental category**. Each file provides the individual genes responding exclusively in abiotic, biotic, ecotype, chemical, hormone, mutant, nutrient or organ comparisons, respectively. Click here for file

Additional file 8 **Genes regulated specifically in one experimental category**. Each file provides the individual genes responding exclusively in abiotic, biotic, ecotype, chemical, hormone, mutant, nutrient or organ comparisons, respectively. Click here for file

Additional file 9 **Genes regulated specifically in one experimental category**. Each file provides the individual genes responding exclusively in abiotic, biotic, ecotype, chemical, hormone, mutant, nutrient or organ comparisons, respectively. Click here for file

Additional file 10 **Genes regulated specifically in one experimental category**. Each file provides the individual genes responding exclusively in abiotic, biotic, ecotype, chemical, hormone, mutant, nutrient or organ comparisons, respectively. Click here for file

Additional File 11 **Importance of organ type in the response to abiotic stress in Arabidopsis**. Percentage of genes responding to various stresses in either roots, shoots or both. Data corresponds to the AtGenExpress Abiotic Stress series present in the NASCarrays database. The black zone indicates the percentage of genes responding only in roots; the white zone indicates those responding only in shoots, and the black squares region indicates the genes responding in both tissues Click here for file

Additional file 12 **Gene responsiveness**. Gene responsiveness as determined by the Rank Products and fold-change method. Click here for file

Additional file 13 **Housekeeping and hypervariable genes and their methylation status (1)**. List of Housekeeping and hypervariable genes, classified according their methylation status as defined in:*Zhang X, et al: Genome-wide high-resolution mapping and functional analysis of DNA methylation in arabidopsis. Cell 2006, 126(6): 1189–1201*. Gene annotation was provided by the VirtualPlant system . Click here for file

Additional file 14 **Housekeeping and hypervariable genes and their methylation status (2)**. List of Housekeeping and hypervariable genes, classified according their methylation status as defined in: *Zilberman et al: Genome-wide analysis of Arabidopsis thaliana DNA methylation uncovers an interdependence between methylation and transcription. Nat Genet 2007, 39(1): 61–69*. Gene annotation was provided by the VirtualPlant system Click here for file

Additional file 15 **Function of housekeeping and hypervariable genes**. Analysis of over-representation of gene ontology functional terms in housekeeping and hypervariable genes (performed in VirtualPlant – Click here for file

Additional file 16 **Enrichment of cis-acting motifs in the promoter of hypervariable genes**. Frequency distribution of the number of predicted transcription binding sites in the promoter of housekeeping and hypervariable genes and the whole genome. The genes were ranked according to the number of cis-acting regulatory elements in their promoters according to the AGRIS database (X-axis). The points represent the fraction of genes in a bin of 10 motifs. Click here for file

Additional file 17 **Correlation between gene responsiveness as determined by the fold-change method and gene body methylation**. Table listing gene responsiveness as determined by the fold-change method (≥ |2|), and the corresponding frequencies of methylated genes. Click here for file

Additional file 18 **Plot of the correlation between gene responsiveness determined by the fod-change method versus gene body methylation**. This graphs shows the linear correlation between gene responsiveness as determined by fold change ((≥ |2|) and gene body methylation. Click here for file

Additional file 19 **Results of simple regression models, given by experimental category**. Description is as Table 2, see main text. Click here for file

Additional file 20 **ANOVA models for the effect of methylation and TATA-box presence on gene responsiveness, by category of experimental treatment**. The models have the form Y ~ aX + b, where X was a factor encoding the presence or absence of those features as two different levels. We used the 'aov' function in R to fit the model. The F statistic estimates the significance of the contribution of the factors to the response. The differences between the levels of the factors were estimated by the Tukey procedure, using the 'glht' function from the 'multcomp' package in R. This is equivalent to comparing the coefficients of the factors. The Bayesian Information Criteria was calculated in R using the 'BIC' function in the package 'nlme'. This parameter represents the "a posteriori" probability of the model to be true, being maximized when the magnitude of the parameter is minimized. Click here for file

Additional file 21 **Lack of linear correlation between expression levels and gene responsiveness**. Box plot of the signal of a gene across the whole NASC arrays dataset (X-axis) versus gene responsiveness (the number of comparisons in which it is significantly regulated, Y-axis). A simple linear regression model cannot explain the variability in the data (R^2 ^= 0.04). Click here for file
