# Supplementary figures and images for: The rules of gene expression in plants: Organ identity and gene body methylation are key factors for regulation of gene expression in Arabidopsis thaliana
Source: BMC Genomics. 2008 Sep 23;9:438. doi: 10.1186/1471-2164-9-438 (PMC2566314; doi:10.1186/1471-2164-9-438)

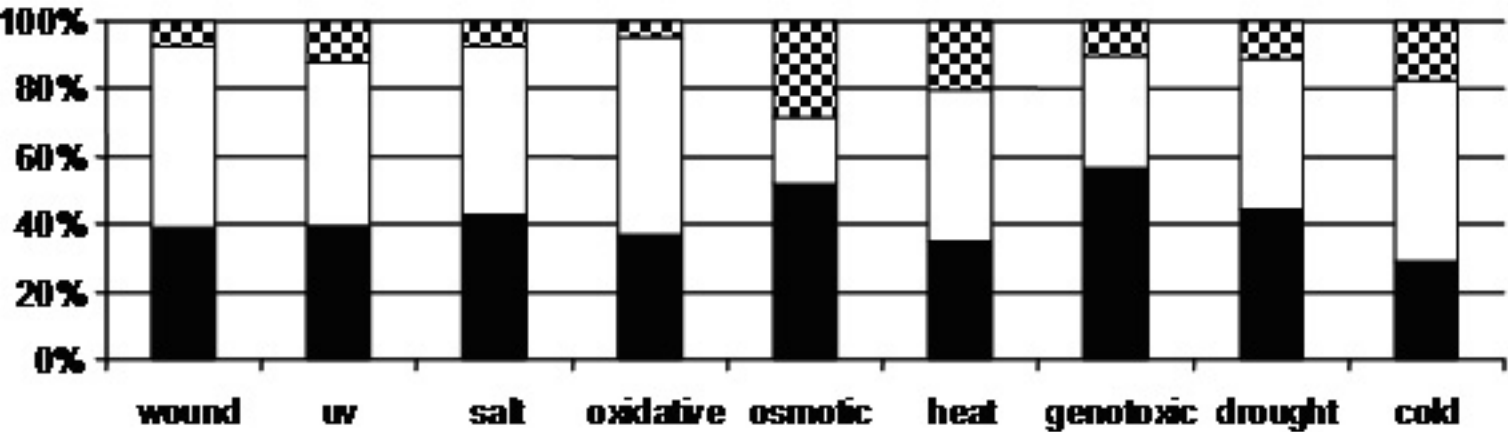

Supplement: Additional File 11 — Importance of organ type in the response to abiotic stress in Arabidopsis. Percentage of genes responding to various stresses in either roots, shoots or both. Data corresponds to the AtGenExpress Abiotic Stress series present in the NASCarrays database. The black zone indicates the percentage of genes responding only in roots; the white zone indicates those responding only in shoots, and the black squares region indicates the genes responding in both tissues [file 1471-2164-9-438-S11.pdf]

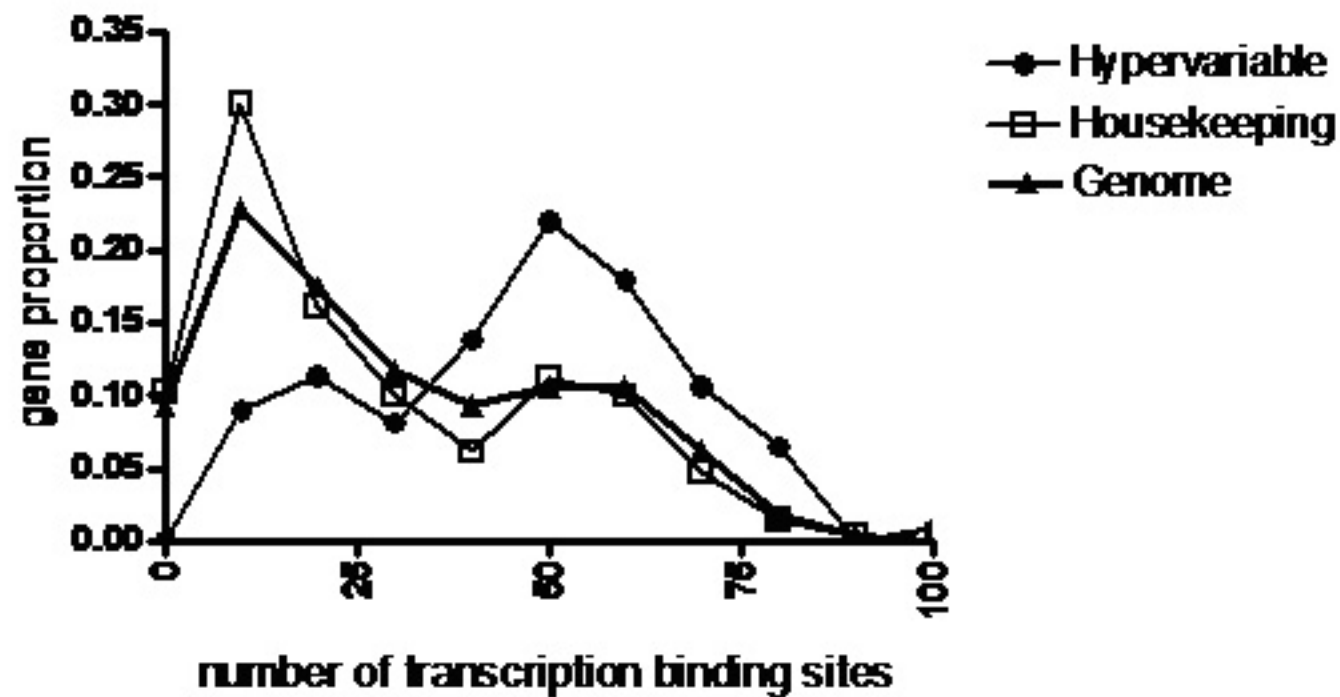

Supplement: Additional file 16 — Enrichment of cis-acting motifs in the promoter of hypervariable genes. Frequency distribution of the number of predicted transcription binding sites in the promoter of housekeeping and hypervariable genes and the whole genome. The genes were ranked according to the number of cis-acting regulatory elements in their promoters according to the AGRIS database (X-axis). The points represent the fraction of genes in a bin of 10 motifs. [file 1471-2164-9-438-S16.pdf]

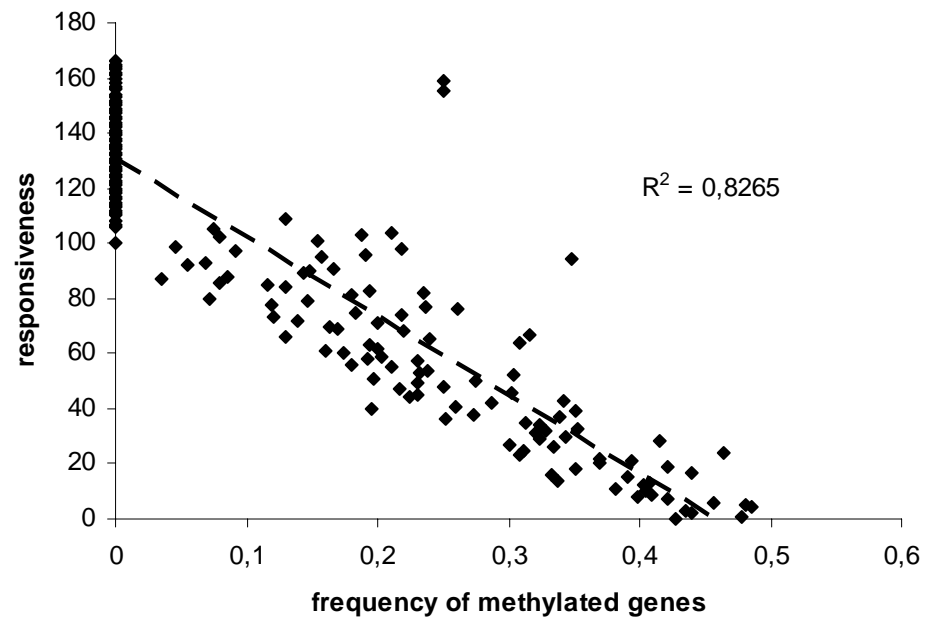

Supplement: Additional file 18 — Plot of the correlation between gene responsiveness determined by the fod-change method versus gene body methylation. This graphs shows the linear correlation between gene responsiveness as determined by fold change ((≥ |2|) and gene body methylation. [file 1471-2164-9-438-S18.pdf]

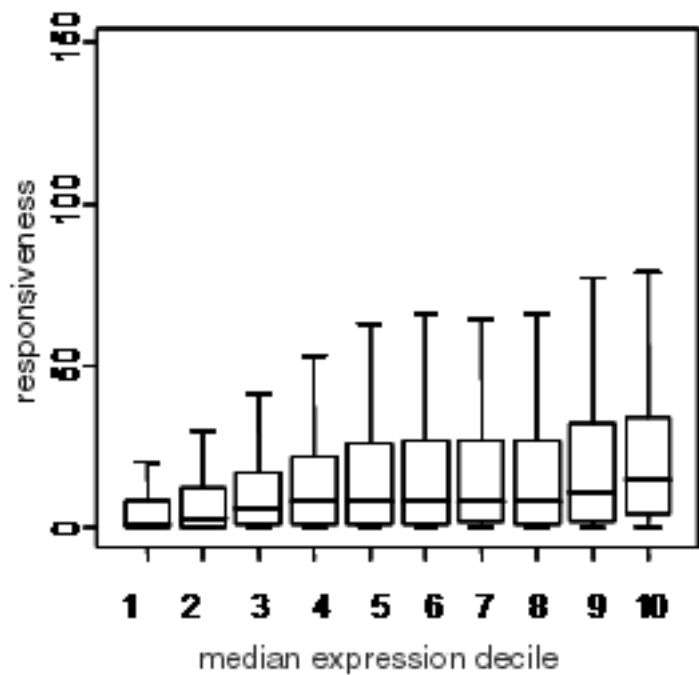

Supplement: Additional file 21 — Lack of linear correlation between expression levels and gene responsiveness. Box plot of the signal of a gene across the whole NASC arrays dataset (X-axis) versus gene responsiveness (the number of comparisons in which it is significantly regulated, Y-axis). A simple linear regression model cannot explain the variability in the data (R2 = 0.04). [file 1471-2164-9-438-S21.pdf]
